# *Polyporus Umbellatus* Protects Against Renal Fibrosis by Regulating Intrarenal Fatty Acyl Metabolites

**DOI:** 10.3389/fphar.2021.633566

**Published:** 2021-02-19

**Authors:** Yan-Ni Wang, Xia-Qing Wu, Dan-Dan Zhang, He-He Hu, Jian-Ling Liu, Nosratola D. Vaziri, Yan Guo, Ying-Yong Zhao, Hua Miao

**Affiliations:** ^1^ Faculty of Life Science and Medicine, Northwest University, Shaanxi, China; ^2^ Division of Nephrology and Hypertension, School of Medicine, University of California Irvine, Irvine, CA, United States; ^3^ Department of Internal Medicine, University of New Mexico, Albuquerque, NM, United States

**Keywords:** lipidomics, chronic renal failure, fatty acid metabolism, *Polyporus umbellatus*, ultra-performance liquid chromatography, mass spectrometry, ergone

## Abstract

**Background:** Chronic renal failure (CRF) results in significant dyslipidemia and profound changes in lipid metabolism. *Polyporus umbellatus* (PPU) has been shown to prevent kidney injury and subsequent kidney fibrosis.

**Methods:** Lipidomic analysis was performed to explore the intrarenal profile of lipid metabolites and further investigate the effect of PPU and its main bioactive component, ergone, on disorders of lipid metabolism in rats induced by adenine. Univariate and multivariate statistical analyses were performed for choosing intrarenal differential lipid species in CRF rats and the intervening effect of n-hexane extract of PPU and ergone on CRF rats.

**Results:** Compared with control group, decreased creatinine clearance rate indicated declining kidney function in CRF group. Based on the lipidomics, we identified 65 lipid species that showed significant differences between CRF and control groups. The levels of 12 lipid species, especially fatty acyl lipids including docosahexaenoic acid, docosapentaenoic acid (22n-3), 10,11-Dihydro-12R-hydroxy-leukotriene C4, 3-hydroxydodecanoyl carnitine, eicosapentaenoic acid, hypogeic acid and 3-hydroxypentadecanoic acid had a strong linear correlation with creatinine clearance rate, which indicated these lipid species were associated with impaired renal function. In addition, receiver operating characteristics analysis showed that 12 lipid species had high area under the curve values with high sensitivity and specificity for differentiating CRF group from control group. These changes are related to the perturbation of fatty acyl metabolism. Treatment with PPU and ergone improved the impaired kidney function and mitigated renal fibrosis. Both chemometrics and cluster analyses showed that rats treated by PPU and ergone could be separated from CRF rats by using 12 lipid species. Intriguingly, PPU treatment could restore the levels of 12 lipid species, while treatment with ergone could only reverse the changes of six fatty acids in CRF rats.

**Conclusion:** Altered intrarenal fatty acyl metabolites were implicated in pathogenesis of renal fibrosis. PPU and ergone administration alleviated renal fibrosis and partially improved fatty acyl metabolism. These findings suggest that PPU exerted its renoprotective effect by regulating fatty acyl metabolism as a potential biochemical mechanism. Therefore, these findings indicated that fatty acyl metabolism played an important role in renal fibrosis and could be considered as an effective therapeutic avenue against renal fibrosis.

## Introduction

Chronic renal failure (CRF) is an irreversible and progressive disease which results in serious complications and its prevalence has risen worldwide (Ortiz et al., 2014; Bansal et al., 2019). CRF indicates that patients have inevitably reached end-stage renal disease and require replacement therapies such as hemodialysis and renal transplantation (Webster et al., 2017). Renal fibrosis including tubulointerstitial fibrosis and glomerulosclerosis are the main pathological features of CRF and is characterized by an excessive accumulation and deposition of extracellular matrix components (Humphreys, 2018; Tang et al., 2019), which are mediated by I- aberrant cellular activities such as fibroblast activation, epithelial-to-mesenchymal transition and monocyte/macrophage infiltration and II- activation of molecules or pathways such as renin-angiotensin system, aryl hydrocarbon receptor, non-coding RNAs, tryptophan metabolism, lipid metabolism, transforming growth factor *ß* (TGF-β)/Smad and Wnt/β-catenin signaling (Chen et al., 2017a; Chen et al., 2019; Edeling et al., 2016; Miao et al., 2020; Zhao, 2013). Although few targeted therapies have been used to delay renal fibrosis, the mentioned-above important findings have revealed the fibrotic cellular and molecular mechanisms underlying the CRF (Nastase et al., 2018; Feng Y. L. et al., 2020).

As fundamental components of the biological membrane lipids play important roles in biological system including composing membrane bilayer, mediating signal transduction, providing functional implementations of membrane proteins and their interactions (Zhao et al., 2014b; Zhao et al., 2015a). In the past several decades, only levels of total cholesterol, triglyceride, low-density lipoprotein cholesterol and high-density lipoprotein cholesterol were determined for investigating the effects of many diseases on lipid metabolism. In 2003, novel lipidomics, a branch of metabolomics, is defined as “the full characterization of lipid molecular species and of their biological roles with respect to expression of proteins involved in lipid metabolism and function, including gene regulation” (Han and Gross, 2003). Lipidomics enables us to systematically identify a wide spectrum of lipid species by using new analytical techniques such as ultra-performance liquid chromatography coupled with high-definition mass spectrometry (UPLC-HDMS) (Zhao et al., 2014c). Based on the metabolomics or lipidomics, several important studies have demonstrated the lipid disorders such as fatty acid metabolism, glycerophospholipid metabolism and sphingolipid metabolism was implicated in CRF by using UPLC-HDMS (Chen et al., 2016; Zhao et al., 2015b; Zhao et al., 2015c).

Traditional Chinese medicines (TCM), as a multi-component drug, can hit multiple targets with multiple components (Chen et al., 2018a; Chen et al., 2018b). In agreement with the holistic thinking of TCM, metabolomics and lipidomics have shown potential in evaluation of therapeutic effects and molecular mechanism of TCM against CRF and renal fibrosis (Chao et al., 2020; Chen et al., 2019b; Dou et al., 2018; Feng et al., 2019; Zhang et al., 2016; Zhang et al., 2018). *Polyporus umbellatus* (PPU) is prepared from the dried sclerotia of *Polyporus umbellatus* (Pers.) Fries, also known as *Grifola umbellata* (Pers.) Pilát (Polyporaceae) (He et al., 2016; Li et al., 2019). It is distributed in Asia, Europe and North America, and is used in China and other countries for centuries to treat a variety of chronic diseases, such as edema, scanty urine vaginal discharge, cloudy painful urinary dysfunction, jaundice, diarrhea, etc (Zhao, 2013). Earlier studies demonstrated that PPU possessed diuretic effect and protected against renal fibrotic effect (Yuan et al., 2004; Zhao, 2013). We have previously reported the significant diuretic activity treated by n-hexane extract of PPU as well as ergosta-4,6,8 (14),22-tetraen-3-one (ergone). Ergone is one of the bioactive steroids that has been isolated from n-hexane extract of PPU (Zhao et al., 2009a; Zhao et al., 2010b). Ergone has been proven to prevent kidney injury and the subsequent renal fibrosis (Zhao et al., 2011b).

In the current study, UPLC-HDMS method was first developed for the determination of intrarenal lipid species in CRF rats induced by adenine. Univariate and multivariate statistical analysis were performed for choosing differential lipid species in adenine-induced CRF rats compared with control rats, and the differential lipid species were identified accordingly. We further demonstrated the intervening effect of n-hexane extracts of PPU and ergone on differential lipid species in CRF rats to reveal the biochemical mechanism of PPU against renal fibrosis.

## Materials and Methods

### Chemicals and Reagents

Adenine (batch Number: A8626, Purity 99.0%) and formic acid solution (ref BCBB6918, purity 50%) were purchased from Sigma Chemical Co., Ltd. LC-grade methanol and acetonitrile were purchased from the Baker Company. Ultra high purity water was prepared using a Milli-Q water purification system. Other chemicals were of analytical grade and their purity was above 99.5%.

### Preparation of N-hexane Extract and Ergone of *Polyporus Umbellatus*

PPU (lot nos 079001) was purchased from Shaanxi Best Enterprise Group Co., Ltd. and was identified by Prof. Feng Wei (National Institutes for Food and Drug Control). A voucher specimen (Z150319) was deposited at the Faculty of Life Science and Medicine, Northwest University, Xi’an, Shaanxi. Crushed PPU (2.3 kg) was extracted three times with methanol at room temperature. The combined extracts were concentrated in vacuum to yield 25.65 g of brown crude extract. The methanol extract of sclerotias was dissolved in 80% MeOH and extracted (partitioned) with n-hexane. 15.48 g of the n-hexane extract were obtained.

The dried n-hexane extract (15 g) was chromatographed on a silica gel column (200–300 mesh). Elution was performed using a solvent mixture of n-hexane/chloroform (2000 ml) with increasing amount of chloroform and similar fractions, identified by TLC G254, were combined to yield 5 main fractions [A, B, C, D, E]. Successive fractions were collected and dried under vacuum using a rotary evaporator. The fraction with n-hexane/chloroform B, C, D and E were tested for their biological activity. Fractions B were further purified by column chromatography, and compound ergone was separated and identified (Supplementary Figure S1) based on our previous study (Zhao et al., 2009a). Ergone purity is 99.9% determined by using high performance liquid chromatography as described previously (Zhao et al., 2009a).

### Animals and Sample Collection

All procedures involving animals were carried out according to the Guide for the Care and Use of Laboratory Animals of the State Committee of Science and Technology of the People’s Republic of China. The protocol was approved by Northwest University institutional animal care and use committee (Permit Number: SYXK 2010-004). Male Sprague-Dawley (SD) rats were obtained from the Central Animal Breeding House of Xi’an Jiaotong University (Xi’an, China). They were maintained at a constant humidity (ca. 60%) and temperature (ca. 23°C) with a 12 h light/dark cycle.

Male Sprague-Dawley rats (body weight 190–210 g) underwent an adaptation period of several days, during which they were fed a commercial feed. Rats aged 6–7 weeks were divided into 9 groups (*n* = 8/group) after measuring of body weight: control group, CRF model group, n-hexane extracted PPU-treated CRF group (CRF + PPU), ergone-treated CRF group (CRF + ERG), and uremic clearance granule-treated CRF group (CRF + UCG). Except for the control group, the rest of the groups were given 200 mg/kg body weight of adenine dissolved in 1% (w/v) gum acacia solution by oral gavage once everyday continuously for 3 weeks, which produced experimental renal failure in the animal for 3 weeks. Control group was similarly given with an equal volume of gum acacia solution. During the adenine of gastric gavage after 3 h, the rats in CRF + ERG group were administered ergone of 46, 92, and 184 mg/kg by oral gavage, respectively. CRF + PPU group were administered n-hexane extracts of 5, 10, and 20 mg/kg by oral gavage, respectively. CRF + UCG group were administered uremic clearance granule of 3600 mg/kg by oral gavage. The group of control and CRF were only administered by oral gavage with the 1% (w/v) gum acacia solution. Body weight was measured daily for all rats. Rats were anesthetized with 10% urethane, and blood samples were obtained by carotid artery cannula, and the left kidney was harvested after *in situ* cardioperfusion*.* Then, the removed kidneys were immediately washed with physiological saline and stored at −80°C for the following histological and lipidomic study.

### Renal Function Evaluation

Serum creatinine was measured by Olympus AU6402 automatic analyzer. Additionally, CCr was calculated to evaluate renal function and the therapeutic effects of PPU and ergone.

### Histological Evaluation

A portion of each fresh kidney was immersed in 10% neutral, buffered formaldehyde solution, then dehydrated, embedded in paraffin, cut in 5 µm sections. Hematoxylin–eosin staining (H&E) and Masson’s trichrome staining were performed as described in detail previously (Dou et al., 2018). Briefly, Sections of 5 μm of paraffin-embedded tissues were mounted on glass slides, rehydrated with distilled water, and stained with Masson’s trichrome method according to Bancroft et al. technique to assess the degree of fibrosis. Fibrotic areas were measured using the Motic Med 6.0 CMIAS pathology image analysis system (Motic, Beijing, China). Data can then be exported directly into Microsoft Excel can be calculated. The data are then subjected to the appropriate statistical analyses. Immunohistochemical staining was performed as described in detail previously (Dou et al., 2018).

### Lipidomic Analysis

The mass data acquired were imported to Markerlynx XS (Waters Corporation, MA, United States) within the Masslynx software for peak detection and alignment. Data analysis methods were shown in our reported literature (Zhao et al., 2013c). The lipidomic procedure including sample preparation, metabolite separation and detection, data preprocessing and statistical analysis for metabolite identification was performed following previous protocols with minor modifications (Zhang et al., 2016; Dou et al., 2018).

### Statistical Analysis

All statistical analyses were accomplished using the software in GraphPad Prism v 6.0 (GraphPad Software, San Diego, CA, United States, RRID: SCR_002798) and SPSS statistical software version 20 (SPSS Inc. IBM, United States). The number of repetitions for each data set was 8, and the results were expressed as mean ± SEM unless otherwise stated.

The acquired raw data from UPLC-HDMS analysis in negative ion modes (Zhao et al., 2013c) were first pre-processed by Markerlynx XS and Progenesis QI (Waters, Manchester, United Kingdom.). Sparse partial least squares-discriminant analysis (sPLS-DA) and principal component analysis (PCA) was performed to discriminate among control, CRF, CRF + PPU, and CRF + ERG groups. A two-tailed unpaired Student's t test is used for the comparison between two groups and statistically significant differences among more than two groups are analyzed using one-way ANOVA followed by Dunnett’s *post hoc tests.* Variables were selected by one-way analysis of variance (ANOVA) with a threshold of *p* < 0.05 in SPSS 20 (SPSS Inc. IBM, United States). Fold change (FC) was calculated based on mean ratios for CRF/controls. Variables were further selected by Mann-Whitney U test with a threshold of *p* < 0.05. The resultant *p* values from ANOVA were further adjusted by a false discovery rate (FDR) based on the Hochberg-Benjamini method. Significantly altered variables were identified. Differential lipids were visualized using heatmap analysis with MetaboAnalyst software (version 4.0). PLS-DA-based receiver operating characteristics (ROC) analysis was performed for the selection of differential variables, and ROC curves were plotted using SPSS. Significantly variables were identified by comparing MS data, MS^E^ fragments, molecular weights and elemental compositions with the available database and reference chemicals. In addition, linear correlation analysis was performed between relative intensity of lipids and CCr.

## Results

### Adenine Led to Declining Renal Function

**FIGURE 1:**
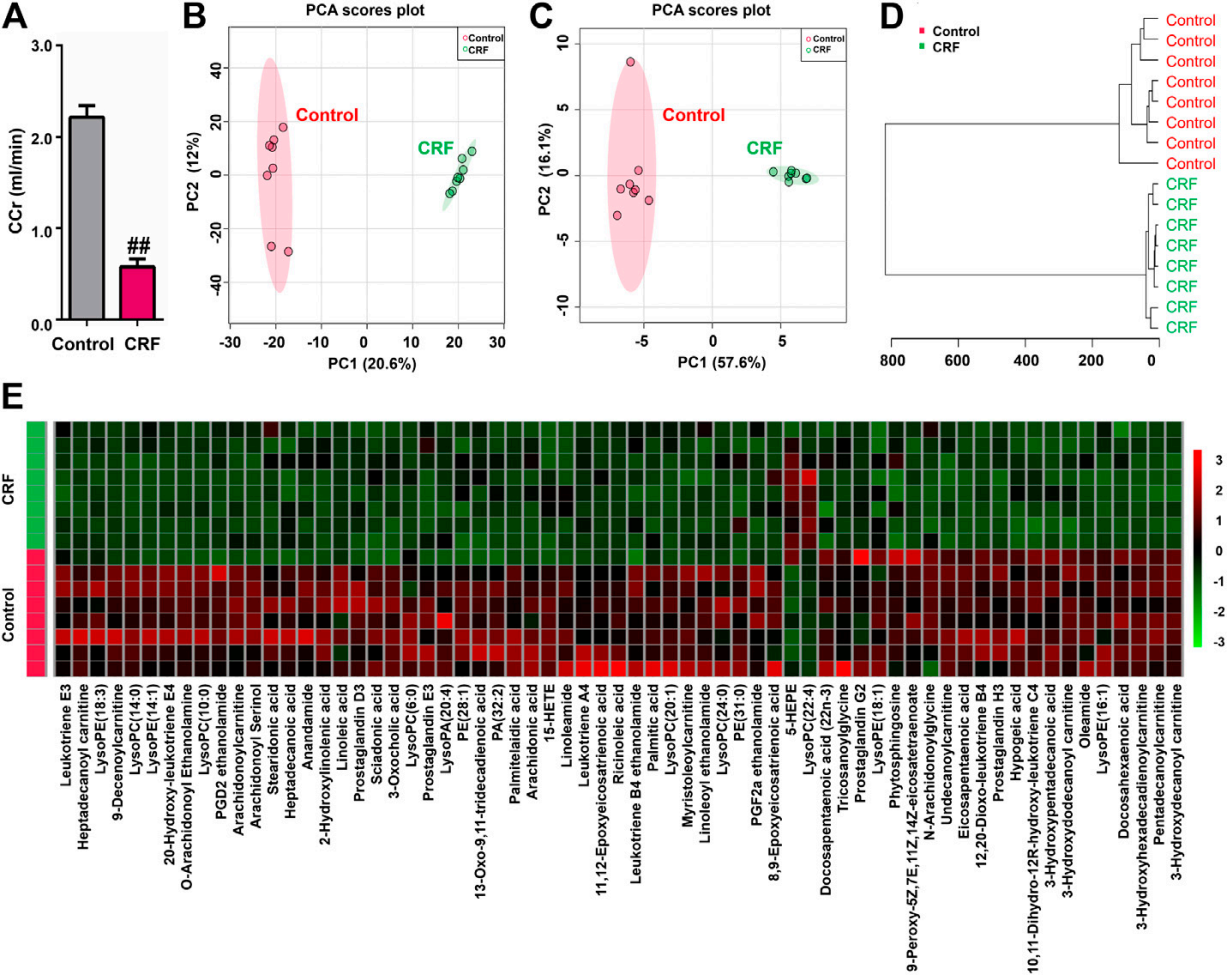
Adenine led to declining renal function and changed lipid metabolic profiling in CRF rats. **(A)** CCr in the control and adenine-induced CRF groups. ^*##*^ *p* < 0.01 compared with control group. **(B)** PCA of two components of 2193 variables from control and adenine-induced CRF groups. **(C)** PCA of two components of 65 lipid species from control and adenine-induced CRF groups. **(D)** Clustering analysis of 65 lipid species form control and adenine-induced CRF groups. **(E)** Heatmap of 65 lipid species between control and adenine-induced CRF groups. Red and green indicate increased and decreased levels, respectively.

In mammalian metabolism, adenine can be oxidized to 2,8-dihydroxyadenine through xanthine dehydrogenase. The very low solubility o f 2,8-dihydroxyadenine causes precipitation in the tubules of the kidney and blocks the tubules. The CCr was significantly decreased in the CRF group compared with control group, which indicated impaired renal function in rats induced by adenine (Figure 1A).

### Adenine Mediated the Changed Lipid Metabolic Profiling in Chronic Renal Failure Rats

The 2193 variables were obtained in the negative ion mode by using UPLC-HDMS. In order to gain an overview of the intrarenal metabolic profiling, PCA was used in the UPLC-HDMS data analysis. Based on 2193 variables, Figure 1B showed the PCA score plot between CRF group and control group. The results showed that CRF group was separated clearly from control group, which indicated that excess adenine led to significantly altered renal metabolism. Therefore, the changes in intrarenal metabolic profiling could reflect the changed renal metabolism.

### Adenine Led to Decreasing the Levels of Lipid Species in Chronic Renal Failure Rats

We further identified the altered metabolites. Initially, 243 variables in negative ion mode have a *p* < 0.05 based on the combination of one way ANOVA and Mann–Whitney *U* test. Subsequently, 114 out of 243 metabolites were selected based on the combination of Bonferroni-adjusted FDR methods and the ROC curves (AUC >0.800). Further, after excluding xenobiotics and different fragment ions from the same metabolites, 65 lipid species were identified based on our previous studies (Table 1) (Zhao et al., 2013a). These lipid species included 49 fatty acyls, 14 glycerophospholipids, 1 steroids and 1 phospholipid could be considered as differential lipid species in kidney issues to distinguish CRF group from control group. 65 lipid species were significantly altered in kidney tissues of CRF rats. Compared with the control group, 63 lipid species were significantly decreased in kidney tissues of CRF group, while two lipid species were significantly increased in kidney tissues of CRF group. In PCA score plot, CRF and control groups were separated clearly by using 65 lipid species (Figure 1C), which is in line with clustering analysis (Figure 1D). The heatmap presented the relative intensity of 65 identified lipid species showing the relative increase (red) or decrease (green) in CRF rats compared with control rats (Figure 1E). The results indicated that intrarenal lipid metabolic profiling was significantly altered in CRF rats.

**TABLE 1:** Identification of differential lipid species in kidney tissues of control and adenine-induced CRF rats.

No	Metabolite	*P* ^a^	*P* ^b^	AUC	FDR^c^	FC^d^	Class
1	12,20-Dioxo-leukotriene B4	1.43E-05	1.55E-04	1.00 E+00	1.01E-02	0.09	Fatty acyls
2	Leukotriene E3	1.02E-03	3.11E-04	9.84E-01	4.04E-03	0.27	Fatty acyls
3	PGF2a ethanolamide	9.13E-04	1.09E-03	9.53E-01	7.07E-03	0.25	Fatty acyls
4	Prostaglandin H3*	5.08E-04	1.09E-03	9.53E-01	6.43E-03	0.26	Fatty acyls
5	11,12-Epoxyeicosatrienoic acid	4.09E-03	1.86E-03	9.30E-01	9.32E-03	0.06	Fatty acyls
6	20-Hydroxy-leukotriene E4	3.32E-04	2.95E-03	9.14E-01	1.01E-02	0.08	Fatty acyls
7	15-HETE	1.14E-03	4.66E-03	9.06E-01	1.26E-02	0.25	Fatty acyls
8	Prostaglandin D3	1.59E-03	4.66E-03	8.98E-01	1.08E-02	0.21	Fatty acyls
9	DHLC4*	6.73E-03	1.04E-02	8.75E-01	1.88E-02	0.48	Fatty acyls
10	PGD2 ethanolamide	2.71E-03	1.04E-02	8.75E-01	1.28E-02	0.31	Fatty acyls
11	Prostaglandin E3	7.55E-03	1.48E-02	8.59E-01	1.65E-02	0.29	Fatty acyls
12	Prostaglandin G2	2.39E-02	2.07E-02	8.44E-01	2.20E-02	0.08	Fatty acyls
13	5-HEPE	1.02E-02	3.79E-02	8.05E-01	3.85E-02	3.22	Fatty acyls
14	Heptadecanoyl carnitine	3.19E-04	3.11E-04	9.84E-01	5.05E-03	0.12	Fatty acyls
15	9-Decenoylcarnitine	2.59E-04	1.86E-03	9.30E-01	8.66E-03	0.01	Fatty acyls
16	Arachidonoylcarnitine	1.20E-03	2.95E-03	9.14E-01	9.14E-03	0.12	Fatty acyls
17	Myristoleoylcarnitine	5.02E-04	6.99E-03	8.91E-01	1.47E-02	0.29	Fatty acyls
18	Pentadecanoylcarnitine*	3.51E-04	6.99E-03	8.83E-01	1.38E-02	0.20	Fatty acyls
19	3-Hydroxydecanoyl carnitine*	6.47E-04	1.04E-02	8.75E-01	1.78E-02	0.31	Fatty acyls
20	3-Hydroxydodecanoyl carnitine*	3.72E-03	1.04E-02	8.75E-01	1.74E-02	0.43	Fatty acyls
21	3-Hydroxyhexadecadienoylcarnitine*	2.79E-04	1.04E-02	8.75E-01	1.69E-02	0.21	Fatty acyls
22	O-arachidonoyl ethanolamine	2.36E-04	1.04E-02	8.75E-01	1.33E-02	0.16	Fatty acyls
23	Undecanoylcarnitine*	1.27E-03	1.04E-02	8.75E-01	1.23E-02	0.35	Fatty acyls
24	PET	8.08E-05	1.55E-04	1.00 E+00	5.05E-03	0.18	Fatty acyls
25	Palmitin acid	1.58E-03	1.09E-03	9.53E-01	7.86E-03	0.48	Fatty acyls
26	Ricinoleic acid	2.60E-03	1.86E-03	9.30E-01	6.73E-03	0.02	Fatty acyls
27	Heptadecanoic acid	5.03E-03	4.66E-03	8.98E-01	1.17E-02	0.32	Fatty acyls
28	Palmitelaidic acid	6.40E-04	4.66E-03	9.06E-01	1.12E-02	0.09	Fatty acyls
29	Sciadonic acid	1.34E-03	6.99E-03	8.83E-01	1.34E-02	0.25	Fatty acyls
30	Docosahexaenoic acid*	1.08E-02	1.00E-02	7.94E-01	1.86E-02	0.53	Fatty acyls
31	13-Oxo-9,11-tridecadienoic acid	8.36E-03	1.04E-02	8.67E-01	1.83E-02	0.33	Fatty acyls
32	3-Hydroxypentadecanoic acid*	3.01E-03	1.04E-02	8.75E-01	1.65E-02	0.39	Fatty acyls
33	Eicosapentaenoic acid*	1.42E-04	1.04E-02	8.75E-01	1.54E-02	0.20	Fatty acyls
34	Hypogeic acid*	7.72E-03	1.04E-02	8.75E-01	1.50E-02	0.42	Fatty acyls
35	Leukotriene A4	2.79E-02	1.04E-02	8.75E-01	1.47E-02	0.27	Fatty acyls
36	Arachidonic acid	6.64E-03	1.48E-02	8.52E-01	1.71E-02	0.21	Fatty acyls
37	8,9-Epoxyeicosatrienoic acid	2.88E-02	2.07E-02	8.44E-01	2.28E-02	0.23	Fatty acyls
38	Docosapentaenoic acid (22n-3)*	2.90E-02	2.07E-02	8.36E-01	2.24E-02	0.47	Fatty acyls
43	Linoleoyl ethanolamide	1.09E-03	1.86E-03	9.38E-01	8.08E-03	0.42	Fatty acyls
39	Tricosanoylglycine	2.01E-02	1.09E-03	9.53E-01	5.89E-03	0.20	Fatty acyls
40	Anandamide	4.28E-03	4.66E-03	8.98E-01	1.21E-02	0.22	Fatty acyls
41	Leukotriene B4 ethanolamide	1.31E-02	1.04E-02	8.75E-01	1.44E-02	0.47	Fatty acyls
42	N-Arachidonoylglycine	2.64E-03	1.04E-02	8.67E-01	1.35E-02	0.31	Fatty acyls
44	Oleamide	1.42E-05	3.11E-04	9.84E-01	2.89E-03	0.22	Fatty acyls
45	Linoleamide	2.71E-03	2.95E-03	9.14E-01	8.72E-03	0.20	Fatty acyls
46	Arachidonoyl serinol	2.69E-04	1.04E-02	8.75E-01	1.57E-02	0.18	Fatty acyls
47	2-Hydroxylinolenic acid	1.05E-03	2.95E-03	9.14E-01	9.60E-03	0.15	Fatty acyls
48	Stearidonic acid	2.44E-02	2.81E-02	8.20E-01	2.90E-02	0.33	Fatty acyls
49	Linoleum acid	6.38E-03	3.79E-02	8.13E-01	3.79E-02	0.00	Fatty acyls
50	LysoPA (20:4)	1.02E-02	1.04E-02	8.67E-01	1.41E-02	0.32	Glycerophospholipids
51	PA (32:2)	3.42E-03	1.04E-02	8.75E-01	1.30E-02	0.33	Glycerophospholipids
52	LysoPC(14:0)	6.33E-05	1.09E-03	9.53E-01	8.84E-03	0.23	Glycerophospholipids
53	LysoPC(6:0)	4.50E-04	1.86E-03	9.30E-01	7.58E-03	0.05	Glycerophospholipids
54	LysoPC(20:1)	8.39E-04	2.95E-03	9.14E-01	8.34E-03	0.11	Glycerophospholipids
55	LysoPC(24:0)	4.14E-03	6.99E-03	8.91E-01	1.57E-02	0.08	Glycerophospholipids
56	LysoPC(10:0)	5.17E-04	1.04E-02	8.75E-01	1.38E-02	0.18	Glycerophospholipids
57	LysoPC(22:4)	5.77E-03	2.81E-02	8.28E-01	2.95E-02	12.86	Glycerophospholipids
58	LysoPE (16:1)	3.91E-05	1.55E-04	1.00 E+00	3.37E-03	0.21	Glycerophospholipids
59	LysoPE (18:3)	2.19E-03	3.11E-04	9.84E-01	3.37E-03	0.18	Glycerophospholipids
60	LysoPE (18:1)	1.36E-03	1.86E-03	9.38E-01	7.13E-03	0.33	Glycerophospholipids
61	LysoPE (14:1)	9.66E-04	6.99E-03	8.83E-01	1.52E-02	0.18	Glycerophospholipids
62	PE (28:1)	4.08E-03	6.99E-03	8.83E-01	1.42E-02	0.31	Glycerophospholipids
63	PE (P-16:0/15:0)	7.70E-03	1.48E-02	8.52E-01	1.68E-02	0.29	Glycerophospholipids
64	Phytosphingosine	4.07E-03	1.04E-02	8.75E-01	1.25E-02	0.38	Phospholipid
65	3-Oxocholic acid	5.27E-04	1.04E-02	8.75E-01	1.61E-02	0.38	Steroids

^a^ *p*-values from one-way ANOVA.

^b^ *p*-values from Mann-Whitney U-test.

^c^ FDR value was obtained from the adjusted *p*-value of FDR correction by Benjamini-Hochberg method.

^d^ FC was obtained by comparing those lipid species in CRF rats with control rats; FC with a value >1 indicated a relatively higher intensity present in CRF rats, whereas a value <1 indicated a relatively lower intensity compared with control rats. Asterisks indicated 12 differential lipid species in kidney tissues based on CCr correlation coefficient R > 0.800.

### Adenine Affected Fatty Acid Metabolism in Chronic Renal Failure Rats

**FIGURE 2:**
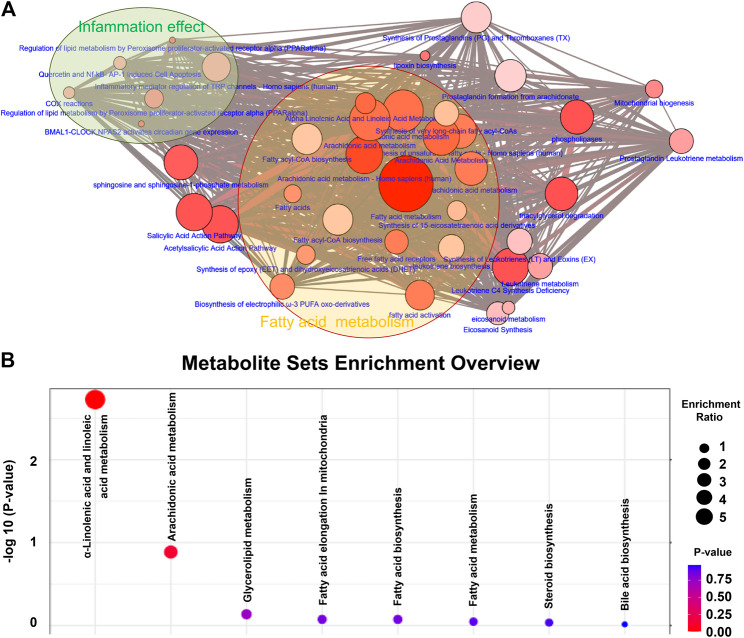
Differential lipid species were associated with fatty acid metabolism. **(A)** Lipid metabolic networks were constructed by using Cytoscape, Reactome, KEGG and NetPath. Adenine-induced CRF were mainly associated with fatty acid metabolism, arachidonic acid metabolism. **(B)** Lipid metabolite sets enrichment overview including α-linolenic acid and linoleic acid metabolism, arachidonic acid metabolism, glycerolipid metabolism, fatty acid elongation in mitochondria, fatty acid biosynthesis, fatty acid metabolism, steroid biosynthesis and bile acid biosynthesis. The size and color of each circle is based on enrichment ratio and *p*-values*,* respectively. Enrichment ratio is computed by hits/expected, where hits indicates observed hits; expected indicates expected hits.

In order to further understand the functional role of differential lipids, we mapped the pathways overrepresented by the identified lipid species from CRF rats, constructed the identified lipid metabolic networks to determine the set that was most enriched by these lipid species. By analyzing known pathways, the image information outcome presented the biological pathway information associated with CRF. The pathway overrepresentation analysis of lipids showed that lipid metabolism was significantly overrepresented in adenine-induced CRF rats, and was related to fatty acid metabolism, arachidonic acid metabolism, fatty acyl-CoA biosynthesis, phospholipases, fatty acid activity, α-linolenic acid and linolenic acid metabolism, synthesis of prostaglandins and thromboxanes and inflammatory mediator regulation of TRP channels etcetera (Figure 2A). These findings suggested that lipid metabolism was severely perturbed in CRF rats.

Metabolite sets enrichment overview of the altered lipid species highlights α-linolenic acid and linoleic acid metabolism, arachidonic acid metabolism and glycerolipid metabolism as being significantly enriched in kidney tissues in CRF vs. control group (Figure 2B). Most of the altered lipid species included fatty acid metabolism, indicating that fatty acid metabolism was severely perturbed in CRF rats based on the combination of lipid metabolic networks and metabolite sets enrichment overview.

### The Dysregulation of Fatty Acids Correlated With Declining Renal Function in Chronic Renal Failure Rats

**FIGURE 3:**
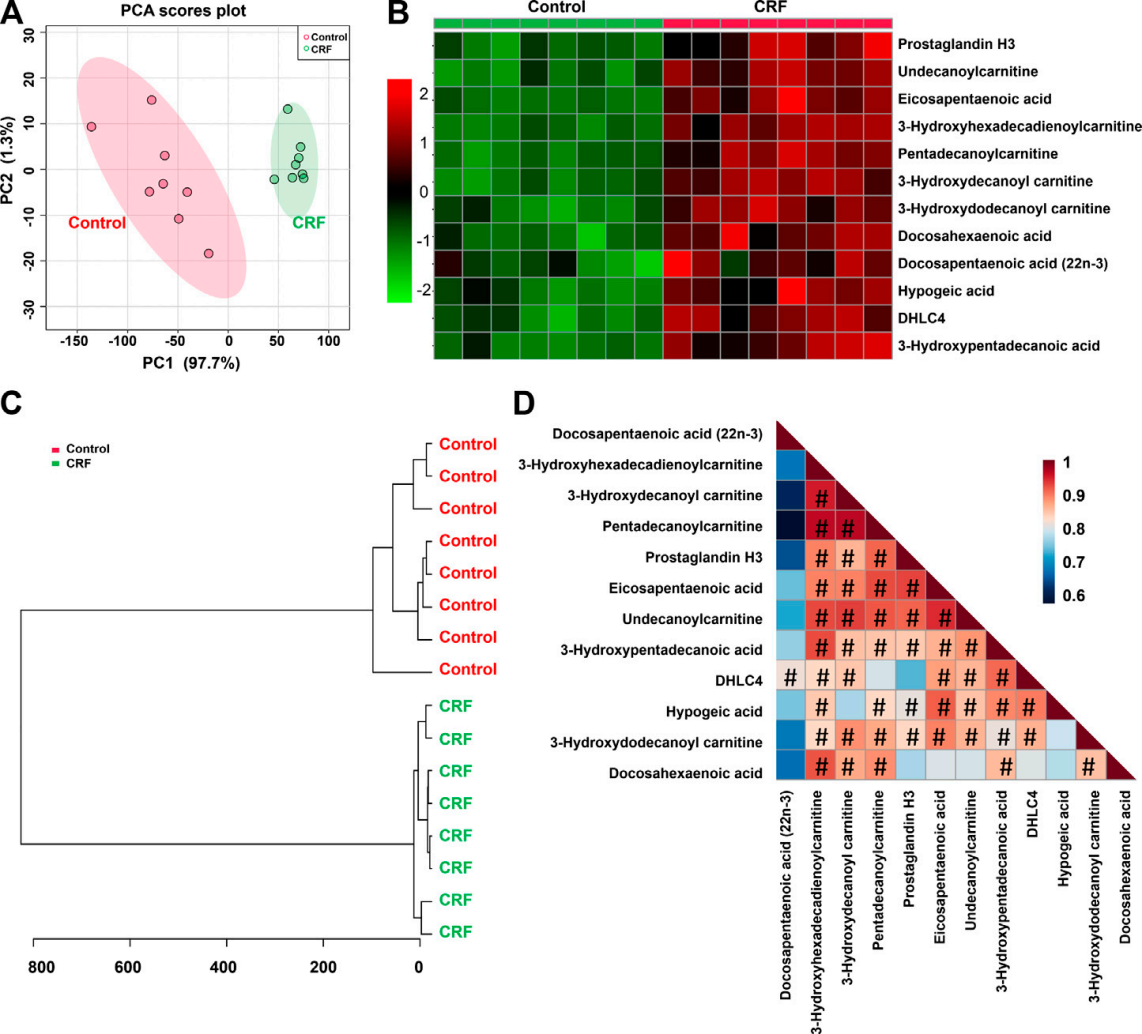
CCr-associated with 12 lipid species were associated with impaired renal function. **(A)** PCA of two components of 12 lipid species from control and adenine-induced CRF groups. **(B)** Heatmap of 12 lipid species between control and adenine-induced CRF groups. Red and green indicate increased and decreased levels, respectively. **(C)** Clustering analysis of 12 lipid species between control and adenine-induced CRF groups. **(D)** Correlation of 12 lipid species among control and adenine-induced CRF groups.

To further verify the potential differential lipid species that may be associated with impaired renal function in CRF rats, we carried out the linear correlation analysis between the levels of each lipid species and CCr. Twelve out of 65 lipid species showed strong linear correlation coefficients (R > 0.800). The twelve lipid species which included prostaglandin H3 (PGH3), 10,11-Dihydro-12R-hydroxy-leukotriene C4 (DHLC4), 3-hydroxydecanoyl carnitine, pentadecanoylcarnitine, 3-hydroxydodecanoyl carnitine, undecanoylcarnitine, hypogeic acid, 3-hydroxyhexadecadienoylcarnitine, 3-hydroxypentadecanoic acid, docosahexaenoic acid (DHA), docosapentaenoic acid (22n-3) (DPA) and eicosapentaenoic acid (EPA) were identified as the top-ranked candidates in the regression model. PCA score plot and clustering analysis indicated that twelve differential lipid species could separate CRF group from control group (Figures 3A,C). The heatmap presents the relative intensity of 12 lipid species showing the relative increase (red) or decrease (green) in CRF rats compared with control rats (Figure 3B). The correlation analysis presented the correlation coefficients of twelve lipid species showing the correlation coefficients more than 0.800 that were indicated by using hash sign (Figure 3D).

**FIGURE 4:**
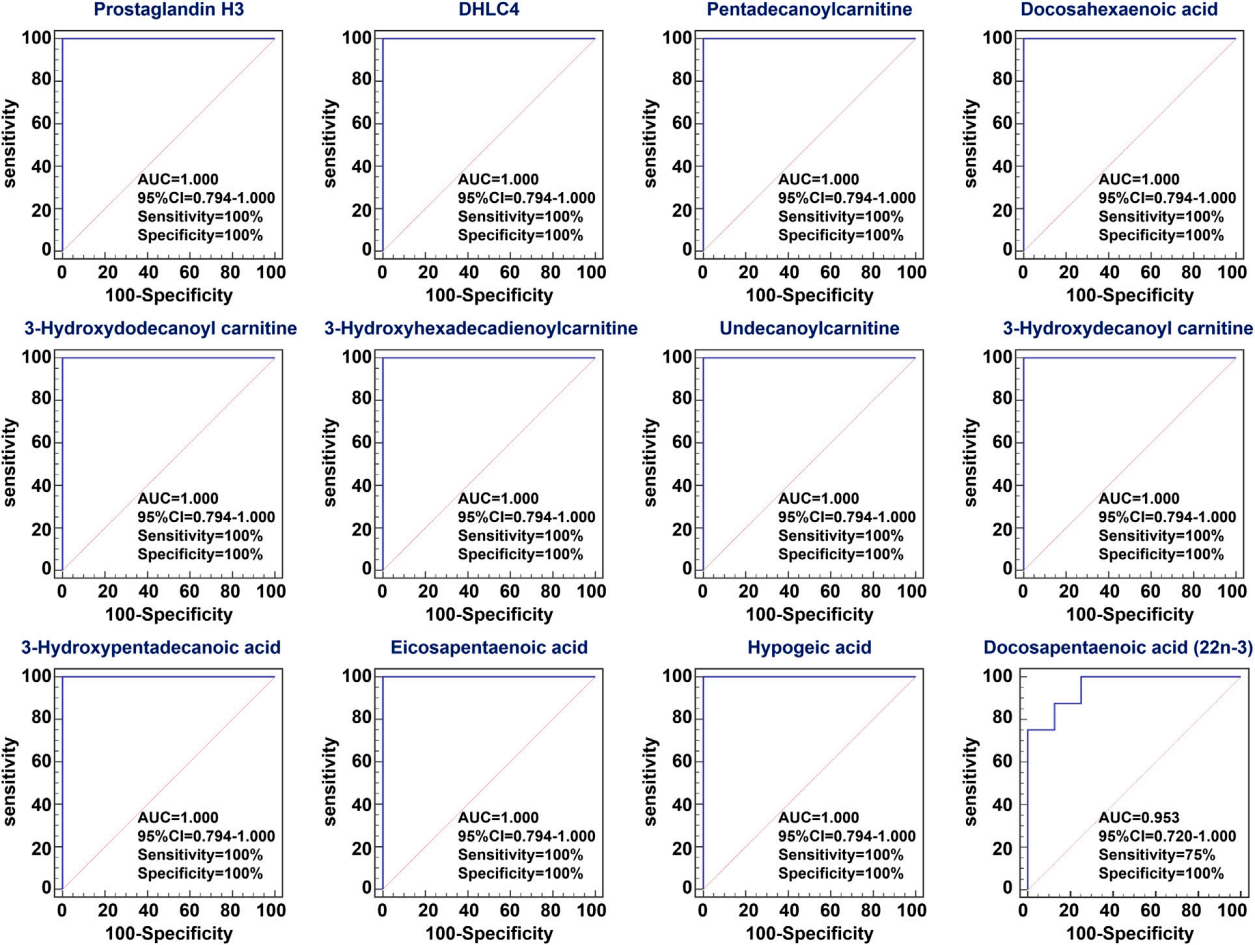
ROC curve of differential lipid species. Analysis of PLS-DA based ROC curves of 12 lipid species in control and adenine-induced CRF groups. The associated AUC, 95% CI, sensitivity and specificity values were indicated.

To assess the predictive performance of 12 lipid species, ROC analysis was performed. ROC results showed that these lipid species have a high AUC value, sensitivity and specificity, indicating that lipid species were robust in distinguishing CRF group from control group (Figure 4). Taken together, these results indicated the significant changes in lipid metabolic profiling and these specific lipid species could be considered to be associated with CRF caused by adenine.

### Treatment With *Polyporus Umbellatus* and Ergone Improved Impaired Renal Function

**FIGURE 5:**
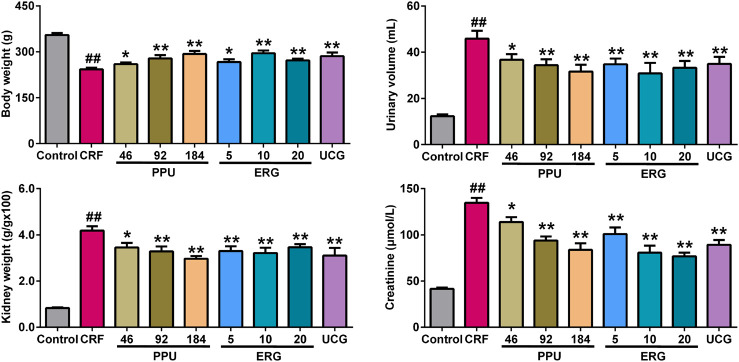
Treatment with PPU and ergone improved impaired renal function. Body weight, urine volume, kidney weight index and serum creatinine levels in the control, CRF, CRF + PPU and CRF + ERG groups. ^*##*^ *p* < 0.01 compared with control group, ^*^ *p* < 0.05, ^**^ *p* < 0.01 compared with CRF group.

The illumination of the underlying disease mechanisms is necessary to establish novel therapeutic strategy for discovering new drug against renal fibrosis. We next assessed the effects of PPU and ergone on impaired renal function and the dysregulation of 12 lipid species in rats with the adenine-induced CRF. Urinary volume, kidney weight index and serum creatinine levels were significantly increased, while body weight was significantly decreased in the adenine-induced CRF group compared with control group (Figure 5). However, urinary volume, kidney weight index and serum creatinine levels were markedly decreased in the different doses of PPU- and ERG-treated groups compared with CRF group. Body weight was significantly increased in the different doses of PPU- and ERG-treated groups compared with the untreated CRF group (Figure 5). These results indicated that impaired renal function was improved after treatment with PPU and ERG. Furthermore, these data indicated that the intervening effect presented a dose-dependent effects of PPU and ergone treatment. Therefore, the PPU of 184 mg/kg dose was chosen as the optimal dose for next experiments. Similarly, the ergone of 10 mg/kg dose showed a stronger intervening effect compared with ergone of 5 mg/kg dose, whereas the intervening effect of 20 mg/kg dose was similar to the effect of 10 mg/kg dose. Therefore, the ergone of 10 mg/kg dose was used for next experiments.

### Treatment With *Polyporus Umbellatus* and Ergone Ameliorated Renal Fibrosis

**FIGURE 6:**
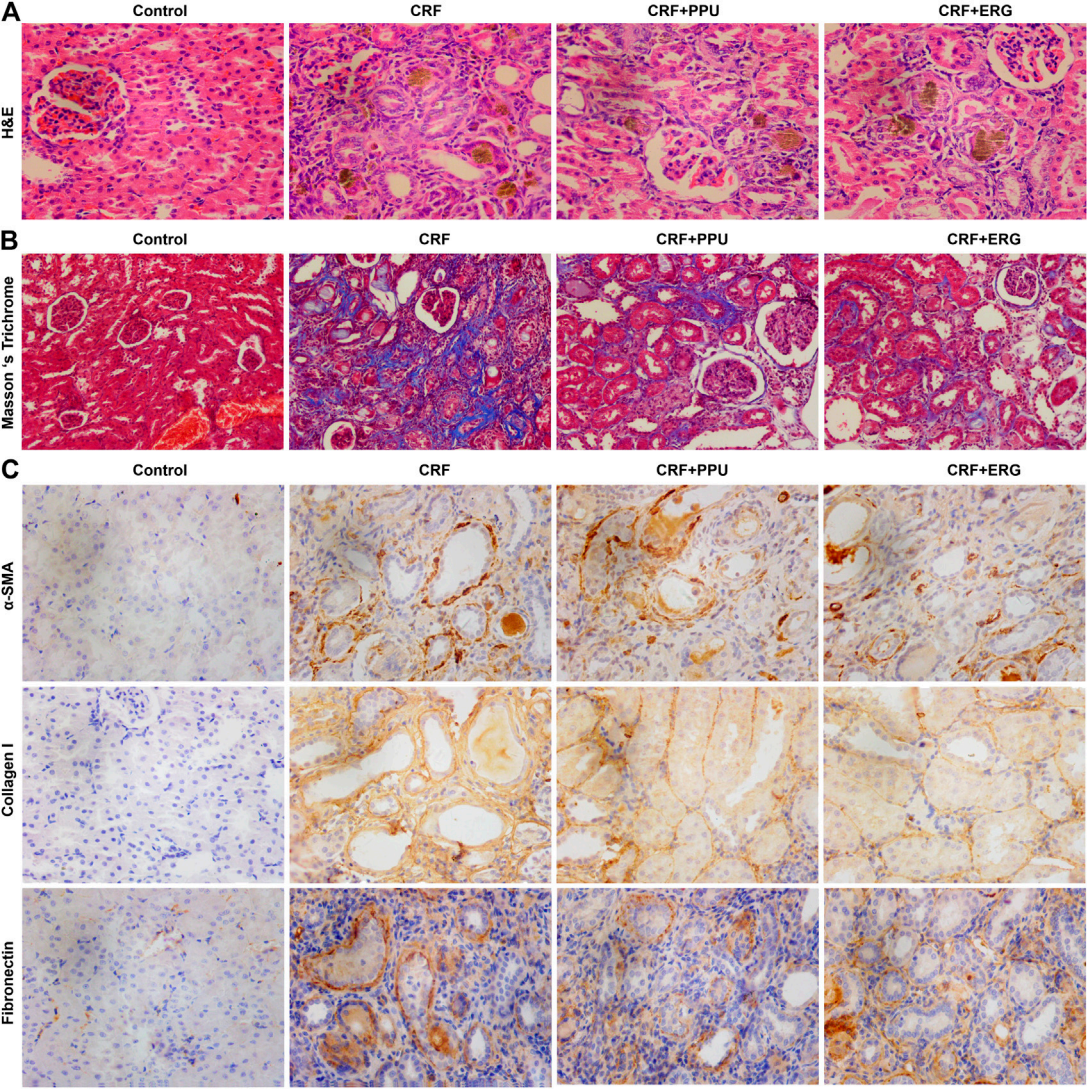
Treatment with PPU and ergone ameliorate renal fibrosis. **(A)** Representative images of H&E stained kidney sections from control, adenine-induced CRF, CRF + PPU, and CRF + ERG groups. Magnification, ×400. **(B)** Representative images of Masson’s trichrome stained kidney sections from control, adenine-induced CRF, CRF + PPU, and CRF + ERG groups. Magnification, ×200. **(C)** Immunohistochemical analyses with anti-α-SMA, collagen I and fibronectin antibodies of rat kidney tissues in the control, adenine-induced CRF, CRF + PPU, and CRF + ERG groups. Magnification, ×400.

H&E straining showed that CRF rats exhibited severe tubular atrophy and dilation, epithelial denudation, inflammatory cell infiltration, granuloma formation and interstitial fibrosis in the kidney tissues (Figure 6A). Masson’s trichrome staining showed severe tubulointerstitial fibrosis in the kidney tissues of CRF rats (Figure 6B). In contrast, these pathological damages were ameliorated in the PPU and ERG treated CRF groups (Figures 6A,B). Adenine administration resulted in a significant increased intrarenal protein expression of fibronectin, collagen I and α-SMA. Treatment with PPU and ergone reduced deposition of these protein compared with the untreated CRF rats (Figure 6C).

### Treatment With *Polyporus Umbellatus* and Ergone Improved Aberrant Fatty Acid Metabolism in Chronic Renal Failure Rats

**FIGURE 7:**
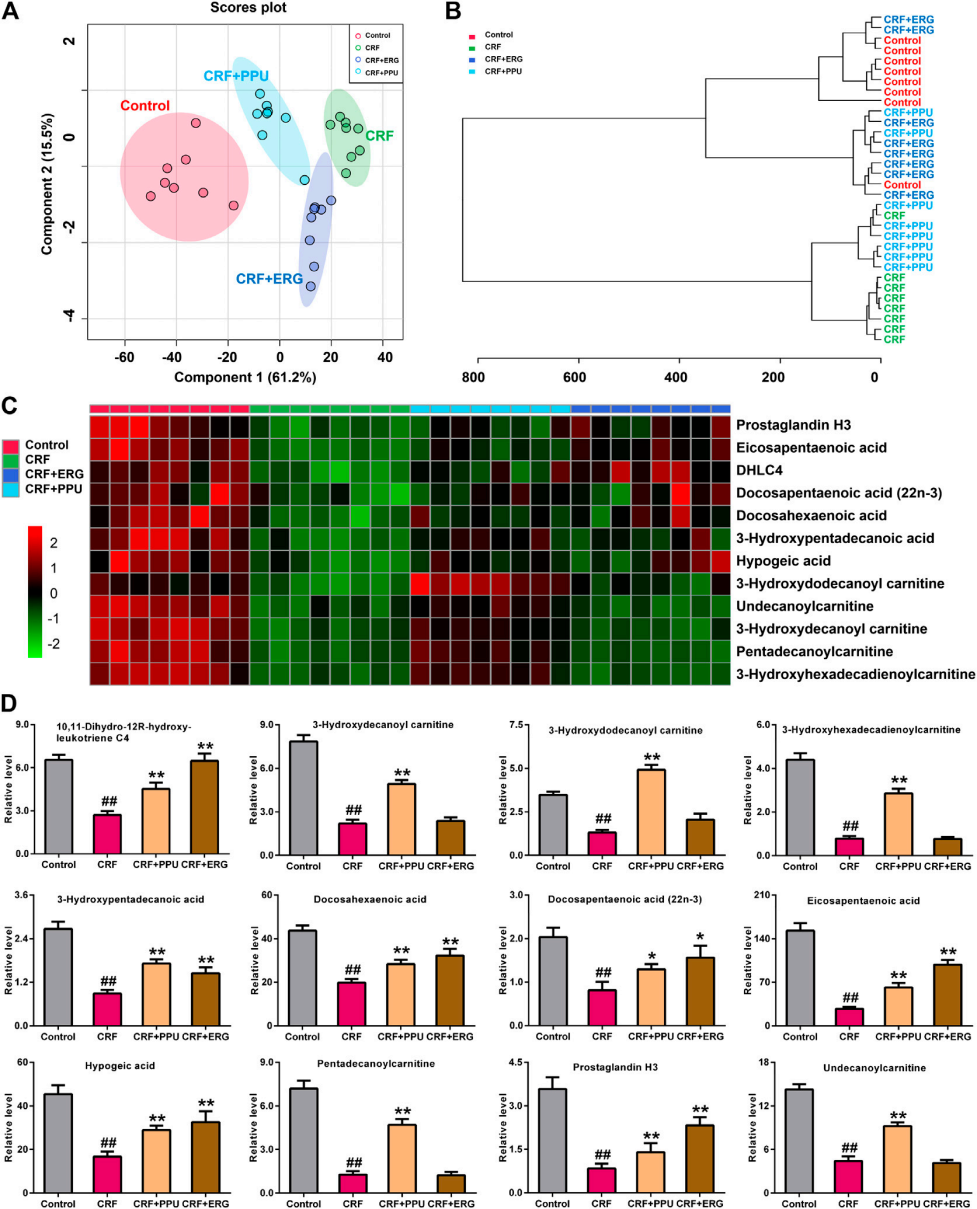
Treatment with PPU and ergone improved aberrant fatty acid metabolism in CRF rats. **(A)** sPLS-DA of two components of 12 lipid species from control, CRF, CRF + PPU, and CRF + ERG groups. **(B)** Clustering analysis of 12 lipid species among control, CRF, CRF + PPU, and CRF + ERG groups. **(C)** Heatmap of 12 lipid species among control, CRF, CRF + PPU, and CRF + ERG groups. Red and green indicate increased and decreased levels, respectively. **(D)** Relative intensity analysis of 12 lipid species among the control, CRF, CRF + PPU, and CRF + ERG groups. ^*#*^ *p* < 0.05, ^*##*^ *p* < 0.01 compared with control group; ^*^ *p* < 0.05, ^**^ *p* < 0.01 compared with CRF group.

The sPLS-DA score plot of 12 lipid species showed PPU- and ERG-treated groups were located between untreated CRF and control groups and further PPU-treated groups was much closer to the control group (Figure 7A), which was consistent with the results of the clustering analysis (Figure 7B) and heatmap analysis (Figure 7C). Relative intensity analysis showed that the levels of 12 lipid species were significant decreases in CRF group compared with control group (Figure 7D). Metabolism of the of 12 lipid species were markedly increased in PPU-treated group compared with CRF group, however, only the levels of fatty acyl metabolites were markedly increased in the ERG-treated group compared with CRF group (Figure 7D). These results indicated that distinct clustering among PPU- and ERG-treated groups and CRF group was achieved in the intervention period, which indicated that lipid metabolic pattern significantly changed after the treatment of PPU and ERG in adenine-induced CRF rats. Intriguingly, the n-hexane extract could improve renal function by improve both fatty acids and carnitine-derived lipid species, while ergone could only targeting improve fatty acids.

## Discussion

Natural products or TCM have been used as a medical therapy for various diseases, including the prevention and treatment of renal diseases since the beginning of civilization (Chen et al., 2018a; Chen Y. Y. et al., 2019; Feng Z. et al., 2020; Hu et al., 2020; Liu et al., 2019; Newman and Cragg, 2016; Yang et al., 2019). A number of natural products such as *Polyporus umbellatus*, *Poria cocos*, and *Alisma orientale* have shown renoprotective properties (Chen et al., 2020; Chen et al., 2019a; Tian et al., 2014; Wang et al., 2018; Wang et al., 2013; Zhao, 2013). PPU is widely used either alone or in combination with other TCM to treat CRF (Ahn et al., 2012). The extract and isolated compounds have shown a wide spectrum of pharmacological activities including antitumor, diuretic and renoprotective effects (Li et al., 2019; Zhao, 2013; Zhao et al., 2012; Zhao et al., 2011a). Its mechanisms of action were found to be related to its anti-inflammatory effect, suppressive epithelial-mesenchymal transition, reconstructed the balance of matrix metalloproteinases and tissue inhibitor of metalloproteinases, and pro-fibrotic and anti-fibrotic factors (Li et al., 2019; Zhao, 2013).

Adenine is a nitrogen whose final metabolite is uric acid. Normally, adenine is efficiently salvaged by adenine phosphoribosyltransferase and is present at very low levels in blood and urine (Zhao et al., 2013b). When the level of adenine is elevated, it can be oxidized to 2,8-dihydroxyadenine. Adenine and 2,8-dihydroxyadenine are excreted in urine. However, due to its very low solubility of 2,8-dihydroxyadenine can lead to its precipitation in renal tubules (Zhao et al., 2014a). Yokzawa et al. reported a new animal model of CRF induced by an adenine rich diet, which can produce metabolic abnormalities resembling chronic renal insufficiency in humans (Yokozawa et al., 1986). In the present study, CRF model was induced by adenine in rats. Adenine consumption led to CRF by inducing aberrant physiological and biochemical parameters and finally renal fibrosis in CRF rats. UPLC-HDMS-based on lipidomics was used to identify differential lipid species in kidney tissues of adenine-induced rats and investigate the effects of n-hexane extract and ergone on differential lipid species to reveal the biochemical mechanisms of their action. We identified 65 differential lipid species in kidney tissues that could distinguish CRF rats from control rats, which suggested that renal injury in CRF rats was associated with altered lipid profile in the renal tissues. Fatty acid metabolism was mainly perturbed in CRF rats based on the combination of lipid metabolic networks and metabolite sets enrichment overview. CCR is a usually index for the indication of kidney function. Twelve differential lipid species were selected based on the linear correlation analysis by using CCr. After n-hexane extract and ergone administration, renal function and fibrosis were significantly improved. These results showed that declining renal function, renal fibrosis and metabolic disturbance of lipids occurred in kidney tissues of rats with adenine-induced CRF, and n-hexane extract and ergone can improve renal function of CRF rats by alleviating renal fibrosis and regulating lipid metabolism.

These differential lipid species in kidneys were mainly related to the pathway of fatty acid metabolism, which showed that fatty acid metabolism of CRF rats was severely disturbed. The n-hexane extract and ergone interfered in fatty acid metabolism in CRF rats. Lipid metabolism plays a key role in CRF, which has been demonstrated by many studies (Chen et al., 2017b; Chen H. et al., 2017; Kang et al., 2015). Normally, fatty acid uptake, oxidation and synthesis are tightly balanced to avoid intracellular lipid accumulation. Significantly decreased levels of polyunsaturated fatty acids, eicosanoid and acylcarnitine were observed in adenine-induced CRF rats, which is consistent with previous reports (Zipser et al., 1983; Kang et al., 2015; Chen et al., 2016). These changes were restored by treatment with n-hexane extract and ergone.

Untargeted lipidomics has been applied to the lipid disorders of the patients with chronic kidney disease (Afshinnia et al., 2016; Duranton et al., 2019; Afshinnia et al., 2020). Afshinnia et al. found that a distinct panel of lipids may improve prediction of progression of chronic kidney disease beyond estimated glomerular filtration rate and urine protein-to-creatinine ratio (Afshinnia et al., 2016). A relationship between polyunsaturated fatty acids and renal inflammation and fibrosis has been hypothesized (Baggio et al., 2005). Several clinical studies have suggested that ω-3 polyunsaturated fatty acid exhibited beneficial effects on patients with end-stage renal disease (Lok et al., 2012). Polyunsaturated fatty acids including EPA, DPA, and DHA are essential ω-3 fatty acid found in fish oils. The levels of EPA, DPA, and DHA were significantly decreased in adenine-induced CRF rats. The ω-3 fatty acid supplementation prevented these changes in lipid species related to inflammation and metabolic lipid disorders. Lee, H et al. found that ω-3 fatty acid supplementation induced the upregulation of six transmembrane protein of prostate 2 protein expression in 5/6 nephrectomized rats, which was associated with an attenuation of inflammation-related markers (Lee et al., 2018). EPA serves as the precursor for the prostaglandin-3 (which inhibits platelet aggregation), thromboxane-3, and leukotriene-5 groups. A diet rich in EPA lowered serum lipid levels, reduced incidence of cardiovascular disorders, prevented platelet aggregation, and inhibited arachidonic acid conversion into the thromboxane-2 and prostaglandin-2 families. DPA is an intermediary between EPA and DHA. As a precursor of prostanoids which was only formed from DPA (Zhao et al., 2013a). Deficiency of DPA in experimental animals caused damage mainly attributable to faulty cellular membranes. Outcomes include sudden failure of growth, lesions of the skin, kidney, and connective tissue, erythrocyte fragility, impaired fertility, and the uncoupling of oxidation and phosphorylation (Halade et al., 2018). Experiments using tubular epithelial cells indicated that inhibition of fatty acid oxidation causes ATP depletion, cell death, intracellular lipid deposition, and dedifferentiation to pro-fibrotic phenotype (Mori et al., 2009). In contrast, restoration of fatty acid metabolism by pharmacological manipulations protected mice from tubulointerstitial fibrosis (Kang et al., 2015)*.* The ω-3 polyunsaturated fatty acids could reverse endothelial dysfunction in chronic kidney disease by improving endothelial nitric oxide synthase function and ameliorating oxidative stress (Zanetti et al., 2017). The lipid profile in patients with CRF is amenable to favourable modification by ω-3 polyunsaturated fatty acids.

PGH3 can be enzymatically converted by platelets into thromboxane A3. PGH3 and thromboxane A3 increased platelet cyclic AMP in platelet-rich plasma and thereby inhibited aggregation by other agonists and suppressed platelet phospholipase-A_2_ activity or events leading to its activation. PGH3 could synthesize PGI3 by blood vessel enzyme. PGI3 is effective coronary vasodilators. They could also inhibit the aggregation of platelet rich plasma and increase the activity of platelet adenylate cyclase. Takeshita A et al. study strongly suggests the potential therapeutic efficacy of recombinant thrombomodulin for the treatment of CRF and subsequent organ failure (Takeshita et al., 2020).

Lipid species including 3-hydroxydecanoyl carnitine, 3-hydroxydodecanoyl carnitine, undecanoylcarnitine and 3-hydroxyhexadecadienoylcarnitine belongs to the family of acyl carnitines, which are organic compounds containing a fatty acid with the carboxylic acid attached to carnitine through an ester bond. Acyl carnitines are essential compounds for fatty acid metabolism (Wang et al., 2019). Several steps are involved in the oxidation of long chain fatty acids by mitochondria. First, fatty acid such as palmitoyl in the presence of CoA, ATP and acyl-CoA synthetase penetrated the outer membrane of the mitochondria and reaches the intermediate space as fatty acid CoA (Smogorzewski et al., 1988). This compound in the presence of carnitine and carnitine palmitoyl transferase is converted into fatty acid-carnitine and CoA (Smogorzewski et al., 1988). The fatty acid-carnitine transverses the inner membrane of the mitochondria, a process which is facilitated by acyl carnitine translocate. In the matrix of the mitochondria, fatty acid-carnitine is again converted into fatty acid CoA and carnitine by carnitine palmitoyl transferase. The fatty acid CoA enters the *ß*-oxidation step. Thus, the inhibition of the oxidation of long-chain fatty acids by n-hexane extract could be interfered by hormone in these steps, while ergone improved the metabolism of fatty acids without affecting the transport of fatty acids to mitochondria. Afshinnia F et al. showed that the increase of saturated C_16_-C_20_ free fatty acid levels, damage *ß*-oxidation of free fatty acid and reverse distribution into complex lipids may be the key mechanism of lipid metabolism changes leading to CKD progression (Afshinnia et al., 2018).

Our current findings have revealed the therapeutic effects and mechanisms of PPU and ergone against renal fibrosis. However, all the experiments evaluating the effects of PPU and ergone were carried out in animal model, which can be seen as a potential limitation. Further interventional effects in patients with CRF should be performed in the future to evaluate the proposed therapeutic effects.

## Conclusion

A lipidomics approach based on UPLC-HDMS and chemometrics method was applied to study the biochemical mechanism of impaired kidney function and renal fibrosis in adenine-induced CRF rats. A clear separation of adenine-induced CRF and control groups was achieved based on identified differential 65 lipid species in kidney tissues. Furthermore, twelve differential lipid species were correlated with declining renal function. Combined with biochemistry and histopathology results, the significantly changes in lipid species were related to perturbation of fatty acid metabolism. The observed changes were reversed by treatment with n-hexane extract and ergone. These findings demonstrated the association of altered intrarenal lipid metabolism with impaired kidney function and therapeutic mechanism of n-hexane extract and ergone against renal fibrosis. Interestingly, inflammation related metabolic pathways have also changed significantly, which is worthy of further study. In combination with traditional therapies, the application of lipidomic profiling throughout the drug discovery and development process and extending into the clinic is likely to lead to improved pharmacotherapy by supporting individualized drug treatment.

## Data Availability

The raw data supporting the conclusions of this article will be made available by the authors, without undue reservation.

## Abbreviations

DHLC4, 10,11-Dihydro-12R-hydroxy-leukotriene C4; CRF, chronic renal failure; CCr, creatinine clearance rate; DHA, docosahexaenoic acid; DPA, docosapentaenoic acid (22n-3); EPA, eicosapentaenoic acid; ergone (ERG), ergosta-4,6,8(14),22-tetraen-3-one; FDR, false discovery rate; FC, fold change; H&E, hematoxylin–eosin staining; MS^E^, mass spectrometry^Elevated Energy^; PPU, *Polyporus umbellatus*; PCA, principal component analysis; PGH3, prostaglandin H3; sPLS-DA, sparse partial least squares-discriminant analysis; TXA3, thromboxane A3; UPLC-HDMS, ultra-performance liquid chromatography coupled with high-definition mass spectrometry.

## References

[B1] Afshinnia F. Rajendiran T. M. Karnovsky A. Soni T. Wang X. Xie D. (2016). Lipidomic signature of progression of chronic kidney disease in the chronic renal insufficiency cohort. Kidney. Int. Rep. 1 (4), 256–268. 10.1016/j.ekir.2016.08.007 28451650 PMC5402253

[B2] Afshinnia F. Rajendiran T. M. Soni T. Byun J. Wernisch S. Sas K. M. (2018). Impaired β-oxidation and altered complex lipid fatty acid partitioning with advancing CKD. J. Am. Soc. Nephrol. 29 (1), 295–306. 10.1681/asn.2017030350 29021384 PMC5748913

[B3] Afshinnia F. Jadoon A. Rajendiran T. M. Soni T. Byun J. Michailidis G. (2020). Plasma lipidomic profiling identifies a novel complex lipid signature associated with ischemic stroke in chronic kidney disease. J. Transl. Sci. 6 (6), 419 33240530 PMC7682927

[B4] Ahn Y. M. Cho K. W. Kang D. G. Lee H. S. (2012). Oryeongsan (Wulingsan), a traditional chinese herbal medicine, induces natriuresis and diuresis along with an inhibition of the renin-angiotensin-aldosterone system in rats. J. Ethnopharmacol. 141 (3), 780–785. 10.1016/j.jep.2012.02.021 22366680

[B5] Baggio B. Musacchio E. Priante G. (2005). Polyunsaturated fatty acids and renal fibrosis: pathophysiologic link and potential clinical implications. J. Nephrol. 18 (4), 362–367. 10.1017/S0029665107005472 16245238

[B6] Bansal N. Zelnick L. Bhat Z. Dobre M. He J. Lash J. (2019). Burden and outcomes of heart failure hospitalizations in adults with chronic kidney disease. J. Am. Coll. Cardiol. 73 (21), 2691–2700. 10.1016/j.jacc.2019.02.071 31146814 PMC6590908

[B7] Chao Y. Gao S. Li N. Zhao H. Qian Y. Zha H. (2020). Lipidomics reveals the therapeutic effects of EtOAc extract of *Orthosiphon stamineus* benth. on nephrolithiasis. Front. Pharmacol. 11, 1299. 10.3389/fphar.2020.01299 32973524 PMC7472562

[B8] Chen H. Cao G. Chen D. Q. Wang M. Vaziri N. D. Zhang Z. H. (2016). Metabolomics insights into activated redox signaling and lipid metabolism dysfunction in chronic kidney disease progression. Redox Biol. 10, 168–178. 10.1016/j.redox.2016.09.014 27750081 PMC5066525

[B9] Chen D. Q. Cao G. Chen H. Liu D. Su W. Yu X. Y. (2017a). Gene and protein expressions and metabolomics exhibit activated redox signaling and wnt/β-catenin pathway are associated with metabolite dysfunction in patients with chronic kidney disease. Redox Biol. 12, 505–521. 10.1016/j.redox.2017.03.017 28343144 PMC5369369

[B10] Chen D. Q. Chen H. Chen L. Vaziri N. D. Wang M. Li X. R. (2017b). The link between phenotype and fatty acid metabolism in advanced chronic kidney disease. Nephrol. Dial. Transplant. 32 (7), 1154–1166. 10.1093/ndt/gfw415 28339984

[B11] Chen H. Chen L. Liu D. Chen D. Q. Vaziri N. D. Yu X. Y. (2017). Combined clinical phenotype and lipidomic analysis reveals the impact of chronic kidney disease on lipid metabolism. J. Proteome Res. 16 (4), 1566–1578. 10.1021/acs.jproteome.6b00956 28286957

[B12] Chen D. Q. Feng Y. L. Cao G. Zhao Y. Y. (2018a). Natural products as a source for antifibrosis therapy. Trends Pharmacol. Sci. 39 (11), 937–952. 10.1016/j.tips.2018.09.002 30268571

[B13] Chen D. Q. Hu H. H. Wang Y. N. Feng Y. L. Cao G. Zhao Y. Y. (2018b). Natural products for the prevention and treatment of kidney disease. Phytomedicine 50, 50–60. 10.1016/j.phymed.2018.09.182 30466992

[B14] Chen L. Cao G. Wang M. Feng Y. L. Chen D. Q. Vaziri N. D. (2019a). The matrix metalloproteinase-13 inhibitor Poricoic acid ZI ameliorates renal fibrosis by mitigating epithelial-mesenchymal transition. Mol. Nutr. Food Res., e1900132. 10.1002/mnfr.201900132 30925007

[B15] Chen L. Chen D. Q. Liu J. R. Zhang J. Vaziri N. D. Zhuang S. (2019b). Unilateral ureteral obstruction causes gut microbial dysbiosis and metabolome disorders contributing to tubulointerstitial fibrosis. Exp. Mol. Med. 51 (3), 1–18. 10.1038/s12276-019-0234-2 PMC6437207 30918245

[B16] Chen D. Q. Cao G. Chen H. Argyopoulos C. P. Yu H. Su W. (2019). Identification of serum metabolites associating with chronic kidney disease progression and anti-fibrotic effect of 5-methoxytryptophan. Nat. Commun. 10 (1), 1476. 10.1038/s41467-019-09329-0 30931940 PMC6443780

[B17] Chen Y. Y. Yu X. Y. Chen L. Vaziri N. D. Ma S. C. Zhao Y. Y. (2019). Redox signaling in aging kidney and opportunity for therapeutic intervention through natural products. Free Radical Biol. Med. 141, 141–149. 10.1016/j.freeradbiomed.2019.06.012 31199964

[B18] Chen H. Wang M. C. Chen Y. Y. Chen L. Wang Y. N. Vaziri N. D. (2020). Alisol B 23-acetate attenuates CKD progression by regulating the renin-angiotensin system and gut-kidney axis. Ther. Adv. Chronic Dis. 11, 2040622320920025. 10.1177/2040622320920025 32547719 PMC7249553

[B19] Dou F. Miao H. Wang J. W. Chen L. Wang M. Chen H. (2018). An integrated lipidomics and phenotype study reveals protective effect and biochemical mechanism of traditionally used *Alisma orientale* juzepzuk in chronic kidney disease. Front. Pharmacol. 9, 53. 10.3389/fphar.2018.00053 29472858 PMC5809464

[B20] Duranton F. Laget J. Gayrard N. Saulnier-Blache J. S. Lundin U. Schanstra J. P. (2019). The CKD plasma lipidome varies with disease severity and outcome. J. Clin. Lipidol. 13 (1), 176–185.e8. 10.1016/j.jacl.2018.07.010 30177483

[B21] Edeling M. Ragi G. Huang S. Pavenstädt H. Susztak K. (2016). Developmental signalling pathways in renal fibrosis: the roles of notch, wnt and hedgehog. Nat. Rev. Nephrol. 12 (7), 426–439. 10.1038/nrneph.2016.54 27140856 PMC5529143

[B22] Feng Y. L. Cao G. Chen D. Q. Vaziri N. D. Chen L. Zhang J. (2019). Microbiome-metabolomics reveals gut microbiota associated with glycine-conjugated metabolites and polyamine metabolism in chronic kidney disease. Cell. Mol. Life Sci. 76 (24), 4961–4978. 10.1007/s00018-019-03155-9 31147751 PMC11105293

[B23] Feng Y. L. Chen D. Q. Vaziri N. D. Guo Y. Zhao Y. Y. (2020). Small molecule inhibitors of epithelial-mesenchymal transition for the treatment of cancer and fibrosis. Med. Res. Rev. 40 (1), 54–78. 10.1002/med.21596 31131921

[B24] Feng Z. Liu W. Jiang H. X. Dai H. Gao C. Dong Z. (2020). How does herbal medicine treat idiopathic membranous nephropathy?. Front. Pharmacol. 11, 994. 10.3389/fphar.2020.00994 32719601 PMC7350518

[B25] Halade G. V. Black L. M. Verma M. K. (2018 Jul - Aug). Paradigm shift - Metabolic transformation of docosahexaenoic and eicosapentaenoic acids to bioactives exemplify the promise of fatty acid drug discovery. Biotechnol. Adv. 36 (4), 935–953. 10.1016/j.biotechadv.2018.02.014 29499340 PMC5971137

[B26] Han X. Gross R. W. (2003). Global analyses of cellular lipidomes directly from crude extracts of biological samples by ESI mass spectrometry: a bridge to lipidomics. J. Lipid Res. 44 (6), 1071–1079. 10.1194/jlr.R300004-JLR200 12671038

[B27] He P. Zhang A. Zhang F. Linhardt R. J. Sun P. (2016). Structure and bioactivity of a polysaccharide containing uronic acid from *Polyporus umbellatus* sclerotia. Carbohydr. Polym. 152, 222–230. 10.1016/j.carbpol.2016.07.010 27516268

[B28] Hu H. H. Cao G. Wu X. Q. Vaziri N. D. Zhao Y. Y. (2020). Wnt signaling pathway in aging-related tissue fibrosis and therapies. Ageing Res. Rev. 60, 101063. 10.1016/j.arr.2020.101063 32272170

[B29] Humphreys B. D. (2018). Mechanisms of renal fibrosis. Annu. Rev. Physiol. 80, 309–326. 10.1146/annurev-physiol-022516-034227 29068765

[B30] Kang H. M. Ahn S. H. Choi P. Ko Y. A. Han S. H. Chinga F. (2015). Defective fatty acid oxidation in renal tubular epithelial cells has a key role in kidney fibrosis development. Nat. Med. 21 (1), 37–46. 10.1038/nm.3762 25419705 PMC4444078

[B31] Lee H. W. Lee S. M. Lee M. H. Son Y. K. Kim S. E. An W. S. (2018). Effect of ω-3 fatty acid on STAMP2 expression in the heart and kidney of 5/6 nephrectomy rat model. Mar. Drugs 16 (11), 398. 10.3390/md16110398 PMC6267584 30360481

[B32] Li H. Yan Z. Xiong Q. Chen X. Lin Y. Xu Y. (2019). Renoprotective effect and mechanism of polysaccharide from *Polyporus umbellatus* sclerotia on renal fibrosis. Carbohydr. Polym. 212, 1–10. 10.1016/j.carbpol.2019.02.026 30832835

[B33] Liu D. Chen L. Zhao H. Vaziri N. D. Ma S. C. Zhao Y. Y. (2019). Small molecules from natural products targeting the Wnt/β-catenin pathway as a therapeutic strategy. Biomed. Pharmacother. 117, 108990. 10.1016/j.biopha.2019.108990 31226638

[B34] Lok C. E. Moist L. Hemmelgarn B. R. Tonelli M. Vazquez M. A. Dorval M. (2012). Effect of fish oil supplementation on graft patency and cardiovascular events among patients with new synthetic arteriovenous hemodialysis grafts: a randomized controlled trial. Jama. 307 (17), 1809–1816. 10.1001/jama.2012.3473 22550196 PMC4046844

[B35] Miao H. Cao G. Wu X. Q. Chen Y. Y. Chen D. Q. Chen L. (2020). Identification of endogenous 1-aminopyrene as a novel mediator of progressive chronic kidney disease via aryl hydrocarbon receptor activation. Br. J. Pharmacol. 177 (15), 3415–3435. 10.1111/bph.15062 32219844 PMC7348091

[B36] Mori T. A. Burke V. Puddey I. Irish A. Cowpland C. A. Beilin L. (2009). The effects of [omega]3 fatty acids and coenzyme Q10 on blood pressure and heart rate in chronic kidney disease: a randomized controlled trial. J. Hypertens 27 (9), 1863–1872. 10.1097/hjh.0b013e32832e1bd9 19705518

[B37] Nastase M. V. Zeng-Brouwers J. Wygrecka M. Schaefer L. (2018). Targeting renal fibrosis: mechanisms and drug delivery systems. Adv. Drug Delivery Rev. 129, 295–307. 10.1016/j.addr.2017.12.019 29288033

[B38] Newman D. J. Cragg G. M. (2016). Natural products as sources of new drugs from 1981 to 2014. J. Nat. Prod. 79 (3), 629–661. 10.1021/acs.jnatprod.5b01055 26852623

[B39] Ortiz A. Covic A. Fliser D. Fouque D. Goldsmith D. Kanbay M. (2014). Epidemiology, contributors to, and clinical trials of mortality risk in chronic kidney failure. Lancet. 383 (9931), 1831–1843. 10.1016/s0140-6736(14)60384-60386 24856028

[B40] Smogorzewski M. Piskorska G. Borum P. R. Massry S. G. (1988). Chronic renal failure, parathyroid hormone and fatty acids oxidation in skeletal muscle. Kidney Int. 33 (2), 555–560. 10.1038/ki.1988.33 3361755

[B41] Takeshita A. Yasuma T. Nishihama K. D'Alessandro-Gabazza C. N. Toda M. Totoki T. (2020). Thrombomodulin ameliorates transforming growth factor-β1-mediated chronic kidney disease via the G-protein coupled receptor 15/Akt signal pathway. Kidney Int. 98 (5), 1179–1192. 10.1016/j.kint.2020.05.041 33069430

[B42] Tang P. M. Nikolic-Paterson D. J. Lan H. Y. (2019). Macrophages: versatile players in renal inflammation and fibrosis. Nat. Rev. Nephrol. 15 (3), 144–158. 10.1038/s41581-019-0110-2 30692665

[B43] Tian T. Chen H. Zhao Y. Y. (2014). Traditional uses, phytochemistry, pharmacology, toxicology and quality control of *Alisma orientale* (Sam.) Juzep: a review. J. Ethnopharmacol. 158 (Pt A), 373–387. 10.1016/j.jep.2014.10.061 25446590

[B44] Wang M. Chen D. Q. Chen L. Cao G. Zhao H. Liu D. (2018). Novel inhibitors of the cellular renin-angiotensin system components, poricoic acids, target Smad3 phosphorylation and Wnt/β-catenin pathway against renal fibrosis. Br. J. Pharmacol. 175 (13), 2689–2708. 10.1111/bph.14333 29679507 PMC6003649

[B45] Wang Y. N. Ma S. X. Chen Y. Y. Chen L. Liu B. L. Liu Q. Q. (2019). Chronic kidney disease: biomarker diagnosis to therapeutic targets. Clin. Chim. Acta. 499, 54–63. 10.1016/j.cca.2019.08.030 31476302

[B46] Wang Y. Z. Zhang J. Zhao Y. L. Li T. Shen T. Li J. Q. (2013). Mycology, cultivation, traditional uses, phytochemistry and pharmacology of *Wolfiporia cocos* (Schwein.) Ryvarden et Gilb.: a review. J. Ethnopharmacol. 147 (2), 265–276. 10.1016/j.jep.2013.03.027 23528366

[B47] Webster A. C. Nagler E. V. Morton R. L. Masson P. (2017). Chronic kidney disease. Lancet. 389 (10075), 1238–1252. 10.1016/s0140-6736(16)32064-5 27887750

[B48] Yang T. Chen Y. Y. Liu J. R. Zhao H. Vaziri N. D. Guo Y. (2019). Natural products against renin-angiotensin system for antifibrosis therapy. Eur. J. Med. Chem. 179, 623–633. 10.1016/j.ejmech.2019.06.091 31279295

[B49] Yokozawa T. Zheng P. D. Oura H. Koizumi F. (1986). Animal model of adenine-induced chronic renal failure in rats. Nephron. 44 (3), 230–234. 10.1159/000183992 3785486

[B50] Yuan D. Mori J. Komatsu K. I. Makino T. Kano Y. (2004). An anti-aldosteronic diuretic component (drain dampness) in *Polyporus sclerotium* . Biol. Pharm. Bull. 27 (6), 867–870. 10.1248/bpb.27.867 15187435

[B51] Zanetti M. Gortan Cappellari G. Barbetta D. Semolic A. Barazzoni R. (2017). Omega 3 polyunsaturated fatty acids improve endothelial dysfunction in chronic renal failure: role of eNOS activation and of oxidative stress. Nutrients. 9 (8), 895. 10.3390/nu9080895 PMC5579688 28820443

[B52] Zhang Z. H. Vaziri N. D. Wei F. Cheng X. L. Bai X. Zhao Y. Y. (2016). An integrated lipidomics and metabolomics reveal nephroprotective effect and biochemical mechanism of Rheum officinale in chronic renal failure. Sci. Rep. 6, 22151. 10.1038/srep22151 26903149 PMC4763304

[B53] Zhang Z. H. Li M. H. Liu D. Chen H. Chen D. Q. Tan N. H. (2018). Rhubarb protect against tubulointerstitial fibrosis by inhibiting TGF-β/smad pathway and improving abnormal Metabolome in chronic kidney disease. Front. Pharmacol. 9, 1029. 10.3389/fphar.2018.01029 30271345 PMC6146043

[B54] Zhao Y. Y. (2013). Traditional uses, phytochemistry, pharmacology, pharmacokinetics and quality control of *Polyporus umbellatus* (Pers.) Fries: a review. J. Ethnopharmacol. 149 (1), 35–48. 10.1016/j.jep.2013.06.031 23811047

[B55] Zhao Y. Y. Xie R. M. Chao X. Zhang Y. Lin R. C. Sun W. J. (2009a). Bioactivity-directed isolation, identification of diuretic compounds from *Polyporus umbellatus* . J. Ethnopharmacol. 126 (1), 184–187. 10.1016/j.jep.2009.07.033 19665537

[B56] Zhao Y. Y. Zhao Y. Zhang Y. M. Lin R. C. Sun W. J. (2009b). Qualitative and quantitative analysis of the diuretic component ergone in *Polyporus umbellatus* by HPLC with fluorescence detection and HPLC-APCI-MS/MS. Pharmazie 64 (6), 366–370. 19618671

[B57] Zhao Y. Y. Cheng X. L. Zhang Y. Chao X. Zhao Y. Lin R. C. (2010a). A fast and sensitive HPLC-MS/MS analysis and preliminary pharmacokinetic characterization of ergone in rats. J Chromatogr. B. Analyt. Technol. Biomed. Life Sci. 878 (1), 29–33. 10.1016/j.jchromb.2009.11.013 19944658

[B58] Zhao Y. Y. Cheng X. L. Zhang Y. Zhao Y. Lin R. C. Sun W. J. (2010b). Simultaneous determination of eight major steroids from *Polyporus umbellatus* by high-performance liquid chromatography coupled with mass spectrometry detections. Biomed. Chromatogr. 24 (2), 222–230. 10.1002/bmc.1277 19572263

[B59] Zhao Y. Y. Shen X. Chao X. Ho C. C. Cheng X. L. Zhang Y. (2011a). Ergosta-4,6,8(14),22-tetraen-3-one induces G2/M cell cycle arrest and apoptosis in human hepatocellular carcinoma HepG2 cells. Biochim. Biophys. Acta-Gen. Subjects 1810 (4), 384–390. 10.1016/j.bbagen.2010.12.005 21241775

[B60] Zhao Y. Y. Zhang L. Mao J. R. Cheng X. H. Lin R. C. Zhang Y. (2011b). Ergosta-4,6,8(14),22-tetraen-3-one isolated from *Polyporus umbellatus* prevents early renal injury in aristolochic acid-induced nephropathy rats. J. Pharm. Pharmacol. 63 (12), 1581–1586. 10.1111/j.2042-7158.2011.01361.x 22060289

[B61] Zhao Y. Y. Shen X. Cheng X. L. Wei F. Bai X. Lin R. C. (2012). Urinary metabonomics study on the protective effects of ergosta-4,6,8(14),22-tetraen-3-one on chronic renal failure in rats using UPLC Q-TOF/MS and a novel MS^E^ data collection technique. Process Biochem. 47 (12), 1980–1987. 10.1016/j.procbio.2012.07.008

[B62] Zhao Y. Y. Cheng X. L. Wei F. Bai X. Tan X. J. Lin R. C. (2013a). Intrarenal metabolomic investigation of chronic kidney disease and its TGF-β1 mechanism in induced-adenine rats using UPLC Q-TOF/HSMS/MS^E^ . J. Proteome Res. 12 (2), 692–703. 10.1021/pr3007792 23227912

[B63] Zhao Y. Y. Feng Y. L. Bai X. Tan X. J. Lin R. C. Mei Q. (2013b). Ultra performance liquid chromatography-based metabonomic study of therapeutic effect of the surface layer of *Poria cocos* on adenine-induced chronic kidney disease provides new insight into anti-fibrosis mechanism. PLoS One 8 (3), e59617. 10.1371/journal.pone.0059617 23555727 PMC3608665

[B64] Zhao Y. Y. Lei P. Chen D. Q. Feng Y. L. Bai X. (2013c). Renal metabolic profiling of early renal injury and renoprotective effects of *Poria cocos* epidermis using UPLC Q-TOF/HSMS/MS^E^ . J. Pharmaceut. Biomed. Anal. 81-82, 202–209. 10.1016/j.jpba.2013.03.028 23670099

[B65] Zhao Y. Y. Chen H. Tian T. Chen D. Q. Bai X. Wei F. (2014a). A pharmaco-metabonomic study on chronic kidney disease and therapeutic effect of ergone by UPLC-QTOF/HDMS. PLoS One 9 (12), e115467. 10.1371/journal.pone.0115467 25535749 PMC4275224

[B66] Zhao Y. Y. Cheng X. L. Lin R. C. (2014b). Lipidomics applications for discovering biomarkers of diseases in clinical chemistry. Int. Rev. Cell Mol. Biol. 313, 1–26. 10.1016/b978-0-12-800177-6.00001-3 25376488

[B67] Zhao Y. Y. Wu S. P. Liu S. Zhang Y. Lin R. C. (2014c). Ultra-performance liquid chromatography-mass spectrometry as a sensitive and powerful technology in lipidomic applications. Chem. Biol. Interact. 220, 181–192. 10.1016/j.cbi.2014.06.029 25014415

[B68] Zhao Y. Y. Miao H. Cheng X. L. Wei F. (2015a). Lipidomics: novel insight into the biochemical mechanism of lipid metabolism and dysregulation-associated disease. Chem. Biol. Interact. 240, 220–238. 10.1016/j.cbi.2015.09.005 26358168

[B69] Zhao Y. Y. Vaziri N. D. Lin R. C. (2015b). Lipidomics: new insight into kidney disease. Adv. Clin. Chem. 68, 153–175. 10.1016/bs.acc.2014.11.002 25858872

[B70] Zhao Y. Y. Wang H. L. Cheng X. L. Wei F. Bai X. Lin R. C. (2015c). Metabolomics analysis reveals the association between lipid abnormalities and oxidative stress, inflammation, fibrosis, and Nrf2 dysfunction in aristolochic acid-induced nephropathy. Sci. Rep. 5, 12936. 10.1038/srep12936 26251179 PMC4528220

[B71] Zipser R. D. Radvan G. H. Kronborg I. J. Duke R. Little T. E. (1983). Urinary thromboxane B2 and prostaglandin E2 in the hepatorenal syndrome: evidence for increased vasoconstrictor and decreased vasodilator factors. Gastroenterology 84 (4), 697–703. 10.1016/0016-5085(83)90133-6 6572162

