# Supplementary figures and images for: Polyporus Umbellatus Protects Against Renal Fibrosis by Regulating Intrarenal Fatty Acyl Metabolites
Source: Front Pharmacol. 2021 Feb 19;12:633566. doi: 10.3389/fphar.2021.633566 (PMC7934088; doi:10.3389/fphar.2021.633566)

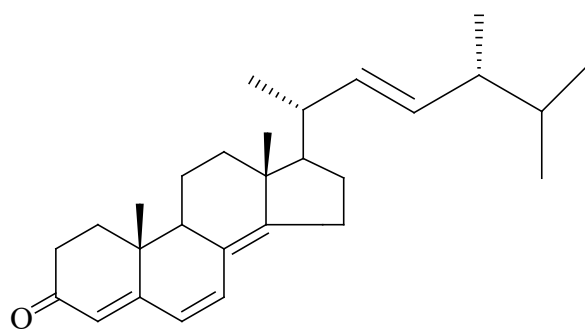

Figure S1 Chemical structure of ergone.

Supplement: Supplementary file 1 [file image1.pdf]
